# Light Waves, Visible and Invisible

**Published:** 1903-09

**Authors:** Frederick Hoyer Millener

**Affiliations:** Buffalo, N. Y.; Surgeon for Diseases of the Nose, Throat and Ear at the German Hospital Dispensary. Member of the Buffalo Academy of Medicine and of the Medical Society of the County of Erie. 724 Main Street


### BUFFALO MEDICAL JOURNAL.

VOL. XLIII.—LIX. SEPTEMBER, 1903. NO. 2.

### ORIGINAL COMMUNICATIONS.

### Light Waves, Visible and Invisible.

BY FREDERICK HOYER MILLENER, M. D., Buffalo, N. Y.

Surgeon for Diseases of the Nose, Throat and Ear at the German Hospital Dispensary. Member of the Buffalo Academy of Medicine and of the Medical Society of the County of Erie.

THE therapeutic potency of light waves, visible and invisible, (the *x*-rays and the ultraviolet light), has of late been vaunted with so much sensational exaggeration in some of the more unscrupulous daily newspapers, that there is some danger of this promising method becoming enveloped in a miasmatic vapor of quackery that may seriously interfere with the development of its practical usefulness and the mischief thus wrought is twofold: false hopes of cure are excited in the minds of sufferers and their friends; and the treatment itself comes to be regarded by medical practitioners as pitch that defiles those who touch it. In this way an element having in it large possibilities of good, may be unjustly discredited in the eyes of both the profession and the public. Hence, it is at the present time especially important that the results obtained by means of the *x*-rays, by practitioners whose work is open to the inspection of their professional brethren, should be fully and accurately recorded with the purpose of showing both what has been attempted and what has been accomplished. Observers of equal honesty receive different impressions from the same phenomena, and even their records of facts take the color of their mental temperament. Inexperience is generally prone to an unfounded optimism; on the other hand, impatience and want of attention to detail, lead to an equally unfounded pessimism. One of the obstacles to the progress of medicine is what may be called the summary mode of dealing with methods on their trial. A method should not be condemned when applied improperly if it fails in unsuitable cases.

For a thorough understanding of the *x*-rays, the ultraviolet light, and other light waves used in medicine, a consideration of the spectrum is necessary. When a beam of sunlight is allowed to pass through a small aperture into a dark room and fall upon a prism, the ray of light passing through the triangular piece of glass is decomposed and, on its emergence upon the other side, is separated into the seven prismatic colors. If the axis of the prism is vertical and the decomposed ray is directed upon a white screen, it will appear as a long narrow ribbon composed of the seven primary colors in which the red will be to the left, followed in order by orange, yellow, green, blue, indigo and violet. If the temperature of these colors be tested by that extremely delicate thermometer, the "thermoelectric pile," it will be found that there is no appreciable heat in the violet, nor in any of the colors until the red is reached, and that tested by the thermometer, the heat increases in the dark space beyond the red ray, which space is known as the infrared, calorific or thermal space. If, on the contrary, a photographic plate is exposed to the action of the prismatic ray, it will be found that the red, orange, and yellow rays have no appreciable effect upon the sensitive coating on the plate, and that it does not commence to darken until it is exposed to the blue light; and further, that the shade gradually increases through the indigo and violet rays, reaching its maximum in the dark space beyond the violet, which space is known as the actinic or ultraviolet space. These waves are of such short length that of course, they cannot be seen. It is in this actinic space beyond the ultraviolet, that the wonderful influence known as the cathode and *x*, or Röntgen rays are found.

The accepted theory of the nature of the forces known as sound, light and heat, is the "undulatory". According to the theory, the entire universe is pervaded by an extremely attenuated mobile gas, known as luminiferous ether, which is imperceptible except when thrown into vibration. A vibration of many hundreds or thousands of waves a second which affects our hearing apparatus, we recognise as sound; a vibration of many millions of waves a second which affects the nerves of feeling, we recognise as heat; a still higher rate of vibration affects the nerves of sight and we recognise it as light. If the vibration comes from a solid body as from the sun, from the fine particles of carbon in a lamp, or from a molten body, the vibrations are of all rates of frequency and all the colors of the spectrum are present, but intimately mixed and blended, we see only the white light.

The discovery of the *x*-rays is due to the investigation of the effect of the electric spark upon the atmosphere. If an electric spark is passed through a tube or vessel containing air, the effect is the same as when passed through the outside air, but if the air in the tube is partially exhausted by means of an air pump, the spark becomes broader, has less of a zig-zag direction and with less noise. If the air is withdrawn as much as possible by the pump, there is no longer any spark, but the whole vessel is filled with a beautiful purplish light. In the Geissler tubes, which are from six inches to several feet in length and from one-half to two inches in diameter, from which the air is almost but not entirely exhausted, hermetically sealed, and fitted with platinum wire at each end, the electric current can be passed through the rarefied atmosphere, producing a brilliant light,—purple, if the tube contains air only; red, if it contains hydrogen, and various other colors with other and different gases.

In the Crookes tubes, a modification of the Geissler tubes, the attenuation of air is carried to so high a degree that it is estimated that a millionth part only of the ordinary atmospheric pressure is present. In seeking the rationale of the phenomena exhibited, it may be said that the particles of the gas contained in the tubes when excited by the electric current vibrate, each particular gas at a definite rate of speed, in a similar manner to the vibration of the strings of a musical instrument, each string vibrating at a certain rate to produce the note to which it is attuned. In considering the *x*-rays, we must remember they are ultra ultraviolet, in fact way beyond the violet; therefore, the waves are so short that they are invisible, so that in some way we must account for the light which we see. It is done as follows: if we regard the tube as a small box filled with small balls, the balls representing the molecules of gas, on shaking the box as the balls cannot move there will be no sound. If some of the balls are removed, the air withdrawn in the case of the tube, allowing the remainder to move freely, on shaking the box the balls will be thrown from side to side. If nearly all of the balls are removed, or the air exhausted, and the box violently shaken, the few balls left will strike against the sides of the box with much force. In the case of the Crookes tubes where the rarefaction is very great, the particles of air are hurled from one end of the tube to the other with such force as to beat the walls of the tube or any object they may strike in the same way as a piece of iron can be made red hot by hammering. These particles of air may be driven against the walls of the tubes with such force as to crack them or melt them by the heat produced. It has been found by Nikola Tesla that particles of air may be forced through the glass. This impact of particles against the sides of the tube imparts to the luminiferous ether a peculiar vibratory motion which is continued through wood, metals, flesh, bone and other opaque objects, and after having passed through them is still capable of affecting a photographic plate and of bringing out fluorescence.

Other rays which are used in medicine and surgery are as follows:

*Ultraviolet.*—If we compare the Röntgen rays with the ultraviolet radiations, we will note some resemblances, but still some notable differences in their physical manifestations:

X-RAYS.

1. Cannot be reflected, refracted, or polarised.
2. Can penetrate and traverse many bodies that will not permit the passage of luminous rays: *e. g.*, wood, aluminum, etc.
3. Will readily traverse the superficial tissues and influence the nutrition of the deeper ones.
4. Will traverse a thick book.
5. Have no appreciable effect on the vitality of bacteria.
6. Will discharge an electroscope, either positively or negatively electrified.
7. Will excite bright green fluorescence in willemite, and induce white phosphorescence in polysulphide of calcium.
8. Rock salt is opaque to *x*-rays.

ULTRAVIOLET RAYS.

1. Can be reflected, refracted and polarised.
2. Will not traverse many bodies that are perfectly pervious to luminous rays, *e. g.*, glass.
3. Will not influence the deeper tissues, nor even the superficial ones, unless they are deprived of their usual blood contents; that is dehematised.
4. Will be stopped by a single leaf of the same book.
5. Will rapidly destroy the vitality of bacteria.
6. Will discharge an electroscope if electrified negatively, but not positively.
7. Will excite bright green fluorescence in willemite, and induce *blue* phosphorescence in polysulphide of calcium.
8. Rock salt is transparent to ultraviolet rays.

The differences here pointed out indicate the fact that we are dealing with agents of totally dissimilar nature, although in certain forms of disease they may both be used to effect the same end; that is, in the treatment of cutaneous affections of an extremely superficial character, while *x*-rays may further be used for the successful treatment of deeper lesions.

*Leonard Rays,* discovered in 1894, have the power of penetrating thin sheets of metal and of producing photographic action as well as discharging electrified bodies. They are also deflected by the magnet. They differ from Röntgen's rays in their penetrative power, for air is relatively opaque to them.

*Becquerel Rays.*—The Becquerel rays are invisible radiations emitted from the salts of the metal uranium, as for example, the nitrate of uranyl and the fluoride of uranium and ammonium. These and other salts of uranium, whether in dark or light, emit a sort of invisible light, which can pass through aluminum and produce on a photographic plate shadows of metal objects. Becquerel's rays possess, like ultraviolet light (though to a lesser degree by reason of their lesser intensity) the property of dis-electrifying charged bodies. These rays are absorbed by air. Water is transparent to them. Metallic solutions are transparent as also are wax and paraffin; uranium glass and red glass 2 mm. thick are fairly opaque. There appears to be no doubt that the uranium rays are a species of extreme ultraviolet light having a wave length certainly less than ten microcentimeters and a frequency certainly greater than 3,000 billions per second.

*Phosphorous Light.*—Professor Thompson has examined the penetrative effect of the pale light emitted by phosphorus when oxidising in moist air. It is accompanied by some invisible rays which will penetrate through black paper or celluloid, but will not pass through aluminum. He found that they emitted rays which, after filtration through card or through copper plates, would act photographically. These rays can be reflected and probably refracted and polarised. He used about a 1,000 fireflies shut up in a shallow box over the screened photographic plate.

*Wiedmann's Rays.*—Professor E. Wiedmann, in 1895, described some rays named by him discharge rays, which are produced in vacuum tubes by the influence of a rapidly alternating electric discharge. They have the property of exciting in certain chemically prepared substances, notably in calcium sulphate containing a small percentage of manganese sulphate, the power of thermo-luminescence. In other words, the substance after exposure to these rays will emit light. When subsequently warmed they are emitted at lower degree of rarefaction than are necessary for producing the *x*-rays. They are emitted from all parts of the path of the spark discharge, but more strongly near the cathode. They are propagated in straight lines but no reflection of them by solid bodies has yet been observed. They are readily absorbed by certain gases, but their production is promoted by hydrogen and nitrogen.

Professor Thompson has recently found two new kinds of cathode rays. One he terms parakathodic, and the other diakathodic ray. The parakathodic ray is produced when ordinary cathode rays strike upon an antikathode, as in the focus tubes. If the vacuum is low, there are emitted from the antikathode, in nearly equal intensity in all directions some rays that closely resemble ordinary cathode rays. They can be deflected electrostatically and magnetically, and can cast shadows of objects on glass walls. If the vacuum is high enough for the production of Röntgen rays some parakathodic rays are produced at the same time. They cause the glass bulb to fluoresce over an obliquely limited region. The diakathodic rays are produced by directing the ordinary cathode rays full upon a piece of wire gauze or upon a spiral piece of wire, which is itself negatively electrified. The ordinary $x$-rays refuse to pass through the meshes of the gauze, but instead there passes through a beam of bluish rays which differ from cathode rays in that they are not directly affected by a magnet. These diakathodic rays can also produce fluorescence of the glass where they meet the walls of the tube, and can cast shadows of intervening objects; but the fluorescence is of a different kind.

*Goldstein's Rays.*—Herr Goldstein has also described some rays apparently closely akin to those just mentioned. If a perforated disk is used as a cathode, there are produced some blue rays which stream back behind the cathode opposite the apertures.

*Radium.*—This new element is about 225 times as heavy as hydrogen. Radium, in its general chemical characteristics, is a member of the same group of elements as calcium. When radium salts, or mixtures rich in radium salts, are brought near the closed eyes or to the temples, a peculiar sensation of light is perceived, not only by those who possess efficient eyes, but by the blind. Radium owes its name to the property which it possesses of emitting spontaneously visible rays in darkness. Radium always emits in darkness a perfectly distinct light. Besides, outside of its own luminescence which is shared by its compounds, it has the power of imparting luminescence to others. This is the reason why substances which become phosphorescent under the action of ultraviolet rays are illuminated in the vicinity of the radium. On the contrary, substances which become phosphorescent in red light remain inert near radium. The degree of luminosity depends evidently on the distance from the radiant source. Moreover, radium transmits to certain metals placed under its influence the property of emitting in their turn radiations. This is called secondary or induced radioactivity; under the radiating action of a particle of radium, a screen of platino-cyanide of barium or of tungstate of calcium is lighted up, as it would be in ultraviolet rays.

The Crookes tubes emit two kinds of rays: cathodic rays lodged in the interior, going from the cathode (—) to the anode (+) and propagated like light in a straight line, but with only half its velocity; and the *x*-rays or Röntgen rays, which only originate at the points where any matter whatever,—solid, liquid or gaseous,—arrests the cathodic rays in their passage. The *x*-rays in the tube focus start, therefore, from the metal plate of the anode, which the cathodic rays have struck, then traverse the glass and are uniformly distributed in every direction near the tube. The radium rays possess at the same time the property of the cathodic rays and of the *x*-rays. Like the cathodic rays the rays which emanate from radium are deflected by the magnet; the cathode rays are totally deflected, the radium rays only partially. A part of the rays remain neutral under the influence of a magnetic field, which leads us to suppose that radium possesses two different species of radiations. The velocity of propagation of radium rays is also half of the velocity of light.

*Finsen's Light.*—Finsen first found if a number of earth worms were placed in an oblong box covered half with red glass and half with blue glass, that the worms will always crawl away from the blue glass and lie under the red. On summing up this and many other experiments he found that all the red or heat rays could do was burn when intense enough, as fire burns. But the actinic rays which do not burn, have other properties that may render them very beneficial or harmful to animal life. Thus, it is the active rays that produce ordinary sunburn,—really not sunburn at all, but an irritation of the skin. He has turned these observations to a practical use in medicine.

*The Peter Cooper Hewitt Light.*—Mr. Hewitt's mercury vapor light is a light in which the red rays are totally absent. It will probably be of value in medicine chiefly as a means of diagnosis, as the appearance of a person under that light is very ghastly, there being no red rays in the light. The result is that any red spot or particle of red on a person or object observed becomes a deep purple. The value of this lamp will thus be evident. What, on an ordinary skin or mucous membrane would appear red, either faintly or more distinct, will now have a distinct purple appearance; therefore, a mild inflammation or rash, may be earlier and more clearly detected.

*Mode of action of the x-rays on diseased tissues.*—The mode of action has not as yet been clearly determined. Their action was at first believed to be mainly bactericidal. This theory had however, to be given up for two reasons. On the one hand, it is very doubtful whether the rays possess the property of destroying microörganisms,—indeed, the majority of investigators believe that they stimulate their growth; on the other, good results have been obtained in diseases, the production and evolution of which microbes play no part whatever. Their effect has also been attributed to the corrosive action of minute platinum particles; to the generation of ozone in the skin and to electric waves given off from the tube. They appear to have marked influence in retarding osmosis. Some authorities think it possible that it is in this property of the *x*-rays that the secret of their biological and therapeutic action lies. By retarding the osmotic processes throughout the system, they cause changes in the intimate condition of cells, constructive or destructive, which must have an influence on the nutrition of the tissues. On the skin the *x*-rays often cause an inflammatory reaction varying in intensity according to the distance and the degree of exhaustion of the tube; the quantity and potential of the current; and the construction of the interruptor, coil, or other apparatus employed.

Neisser compares the action of the rays on the skin to the local action produced by tuberculin. Kaposi held that they acted upon the bloodvessels, producing an alteration in tone, bringing about healing and absorption of the granuloma by fatty degeneration or molecular change. Scholtz experimented on various animals and found microscopic changes, consisting of swelling and edema of the epithelial cells and champing and shrinking of the nuclei, with a clear space in the cell protoplasm. Splitting of the nucleus in cell division was rarely seen, but many of the epithelial cells showed nuclei which seemed to be in process of dividing. There was marked edema of the corium, its fibers being swollen and staining badly, the elastin being more resistant than the collagen. The connective tissue cells were affected in a similar manner to those of the outer layer of skin, as also were the cells of the sweat glands, the hair follicles and the intima of the large bloodvessels. There was abundant inflammatory infiltration of cells, chiefly leucocytes. Masses of leucocytes were present beneath the epidermis; the mast cells in the corium were increased. Toward the center lesions there were superficial vesicles in the stratum corneum. Scholtz concluded that the rays cause a slow degeneration of the cellular elements of the skin, both of the epidermis and corium, the nucleus as well as the protoplasm being affected. There is also a less marked degeneration of the fibrous elements. As soon as the cellular degeneration reaches a certain degree, an inflammatory reaction occurs in which the bloodvessels become dilated and the usual phenomena of inflammation take place—namely, an extravasation of serum and leucocytes. The latter seem to act as phagocytes and completely destroy the degenerated cells. The microscopic changes caused by the action of the *x*-rays on the tissues are numerous, interesting and varied.

*The physiological properties of radium.*—The physiological properties of radium rays are especially remarkable. While the prolonged application of the *x*-rays produces on the skin a simple erythema, perhaps the falling out of the hair or even a burn, radium produces a veritable burn,—an almost intractable burn. It yields only to treatment of months. M. Becquerel was accidentally the victim of these deadly rays, by carrying in his pocket a tube containing a few decigrammes of slightly active radiant matter. M. Curie, voluntarily in the cause of science, devoted himself with true heroism to their cruel attack and sacrificed a part of the skin of his arm. The direct contact of radium causes in a short time a dropping off of the skin followed by severe and persistent pain. The vegetable world is as susceptible as man. The seeds of mustard and garden cress, exposed to the corrosive influence of radium lose their germinating power. This is wonderful, but not so astonishing as the fact that all these different effects, some more singular than others, are produced by a single particle of metal through a double covering of glass, without this magic power being diminished by time. It appears from reliable calculations that with radium the loss of substance caused by the radiating energy for one cubic centimeter, would be one milligramme in a thousand million years.

*How to achieve results by means of the x-rays.*—It is highly important that the result of the *x*-rays treatment should be emphasised by a full and detailed publication. There is undoubted evidence that the benefit to be derived from the *x*-rays is almost illimitable. The great aim of those who adopt this method of treatment is to endeavor to utilise to the fullest the healing qualities and power of the rays. Keen observation and sound judgment are essential and we should have no hesitation in asserting that the success obtained is in proportion to the exercise of these faculties. The results already achieved are of an excellence and endurance sufficient to give substantial indication of what one may expect from the rays in, let us hope, the near future.

*Attention to detail.*—The character of the coil, the voltage used, the condition of the tube, the distance of the patient from the instrument, the duration and frequency of the sittings and the intervals between each; and, also, whether or not, any other treatment is applied. These are details which are many times overlooked, but are of great importance. No hard and fast rule can be adopted, but the effect of the rays can be applied to individual cases and regulated to suit the various stages of a disease, by strict attention to the above injunction. This applies more specially to the condition of the tubes and to the limitation or extension according to requirements of the exposure given the patient.

Different tubes have different capabilities, and a good tube usually comes only by careful nursing and attention. There are many who place too great reliance on the length of the sitting. In many articles I have read the period of exposure adopted by the writer was stated to be fröm ten minutes to an hour every day. This is not the best way. It should always in using these waves be the object to obtain as rapidly as possible a tangible result consistent with safe treatment, because we do not know all of the properties of the light we are using.

*Rays and operation compared.*—The advantages of the $x$-rays over operative treatment are principally in effect. After healing they leave a soft, pliable skin, devoid of contraction, and the face is not disfigured by unsightly scars, which are the inevitable consequences of the use of the knife. The risk from anesthetics is obviated and the patient cured without pain or shock. It is an established fact that in many skin diseases the application of the $x$-rays abolishes the necessity for operation. Those under treatment can also at the same time carry on their ordinary avocations. Many who fear the danger of operation hesitate and in this way the disease is spread and becomes more difficult to cope with. This is a general and broad view to take of the question and its lesions.

REFERENCES.

John MacIntire, British Medical Journal, June 6, 1903.
Malcolm Morris, British Medical Journal.
Works by Curie, Professor Thompson, Crookes and others.

724 Main Street.

